# Limitations of mammography in the diagnosis of breast diseases compared with ultrasonography: a single-center retrospective analysis of 274 cases

**DOI:** 10.1186/s40001-015-0140-6

**Published:** 2015-04-21

**Authors:** Hong Zhao, Liwei Zou, Xiaoping Geng, Suisheng Zheng

**Affiliations:** Department of Radiology, The Second Affiliated Hospital of Anhui Medical University, 678 Furong Road, Hefei, Anhui 230000 China; Department of Surgery, The Second Affiliated Hospital of Anhui Medical University, 678 Furong Road, Hefei, Anhui 230000 China

**Keywords:** Breast disease, Mammography, Ultrasound, Breast cancer, Surgery

## Abstract

**Background:**

The aim of this study is to compare X-ray mammography (MG) and ultrasonography (US) in the diagnosis of breast diseases in Chinese women.

**Methods:**

We retrospectively analyzed X-ray mammograms of 274 patients with US and surgical/pathological results of breast diseases diagnosed at The Second Affiliated Hospital of Anhui Medical University (Hefei, China) between March 2011 and November 2014. The MG and US data were compared to surgical records using the results from post-surgical pathological examinations as the gold standard.

**Results:**

The overall sensitivity, specificity, accuracy, false-positive, false-negative, positive predictive value, and negative predictive value for the detection of breast cancer were 88.5%, 57.9%, 73.7%, 42.1%, 11.5%, 69.2%, and 82.5%, respectively, for MG and 95.9%, 66.7%, 81.8%, 33.3%, 4.1%, 75.5%, and 93.8%, respectively, for US. Of the 274 cases, lesion size by MG agreed with surgery in 133 (48.5%) patients compared with 216 (78.8%) by US (*P* < 0.01). Lesion location by MG agreed with surgery in 146 (53.3%) patients compared with 257 (93.8%) by US (*P* < 0.01). These values were then stratified according to age, menstrual status, breast density, and breast volume, and the agreement rates of MG with surgery were lower than that of US (all *P* < 0.01), except when the lesion size was >5 cm (*P* > 0.05).

**Conclusions:**

US was better than MG in the preoperative evaluation of breast diseases of Chinese women. These results suggest that US could be more useful for detecting breast lesions in China, especially for younger women with dense breasts.

## Background

Breast diseases, both benign and malignant, affect many women worldwide. To enhance early detection, women are encouraged to undergo routine screening by mammography (MG) [1]. Breast density represents the proportion of different tissue types within a woman’s breast. Specifically, breast and connective tissues are denser than fat, and this difference is apparent by MG. When breast density is high (that is, when there is a greater amount of breast and connective tissues compared with fat), mammograms are more difficult to interpret because a lesion may be shadowed by the dense tissues. Moreover, research has shown that women with high breast density are at increased risk of developing breast cancer [2]. Breast density varies by race, and many Chinese women have dense- or intermediate mixed-type breast density [3]. Thus, MG may fail to accurately identify tumors within this population. In some countries, doctors have begun to implement alternative methods for women with dense breasts. Such measures include the use of ultrasonography (US) and magnetic resonance imaging (MRI) [4,5]. MRI is a useful tool to assess breast diseases and has been shown to have a higher sensitivity than MG [4,5]. However, MRI is expensive and waiting lists are often long, limiting its use in underdeveloped areas of China. In contrast, US might be more accurate than MG and is cheaper than MRI for the preoperative evaluation of breast diseases in women [4,5].

Therefore, the present study aimed to retrospectively analyze MG and US of 274 patients with surgical pathology-confirmed breast diseases in the diagnosis of breast diseases in Chinese women, to compare the diagnosis value of MG and US, and to establish an optimal modality of breast diseases in underdeveloped areas of China.

The results of the present study could identify the limitations of MG in the diagnosis of breast diseases in Chinese women, especially in those with high-density and relatively small breasts.

## Methods

### Patients

Two hundred seventy-four consecutive female patients diagnosed with breast diseases and who underwent surgery at The Second Affiliated Hospital of Anhui Medical University (Hefei, China) from March 2011 through November 2014 were included in the present study. Inclusion criteria were as follows: 1) presence of a breast lesion on imagery; 2) the lesion underwent surgery; 3) underwent preoperative MG and US before; 4) and lesion was confirmed by postoperative pathology. Women were excluded if they had undergone only MG or US. This retrospective study was approved by the Institutional Review Board of The Second Affiliated Hospital of Anhui Medical University. The need for individual consent was waived by the committee because of the retrospective nature of the study.

### MG and US assessment

MG and US were both performed 2 weeks before surgery. Mediolateral oblique and craniocaudal digital MG of the breast were performed using a molybdenum-rhodium target full-field digital MG system (Senographe 2000D, General Electric, Pittsburgh, PA, USA). If required, additional MG views were obtained. An automatic exposure factor was used, and adequate pressure was applied on the breast. All MG examinations were read by two radiologists who were blinded to the patient’s identity and medical background. The imaging interpretation was based on the American College of Radiology (ACR) BI-RADS (Breast Imaging Reporting and Data System) lexicon [6]. Breast lesions were classified into six categories according to the lesion margin and calcification status: BI-RADS 0 = unsatisfactory MG, and additional imaging evaluations are needed; BI-RADS 1 = negative, no abnormality on MG; BI-RADS 2 = benign findings, presence of definite benign lesions without any signs of malignancy; BI-RADS 3 = probably benign lesions, including uncalcified lump with negative palpation and clear boundary and focal, asymmetric, clustering, round or dot-like calcifications, and a follow-up in a short time frame is suggested; BI-RADS 4 = suspicious abnormality without typical signs of malignancy, including palpable, solid lumps with some clear margins, palpable complex cysts, palpable abscess, solid mass with irregular shape and infiltrating margin, and newly emerging clustered, tiny, polygonal calcifications, and biopsy should be considered; BI-RADS category 5 = highly suggestive of malignancy and appropriate actions should be taken. The total breast density was classified into ACR levels 1 to 4 [2]: level 1, almost entirely fatty; level 2, scattered fibroglandular densities; level 3, heterogeneously dense; and level 4, extremely dense. In the present study, density levels 1 to 2 were defined as low density, and levels 3 to 4 were defined as high density. The volume of the breast was measured using the formula proposed by Kalbhen *et al*. [7,8]: breast volume = π/4 × (*W* × *H* × *C*), where *W* is the breast width, *H* is the breast height, and *C* is the compression thickness in craniocaudal MG.

US examination was performed using a color Doppler US device (PHLIPS iu22, Philips, Best, The Netherlands) with a probe frequency of 10 to 18 Hz. All US examinations were performed with the patient in the supine position for the medial parts of the breast and in the contralateral posterior oblique position with arms raised for the lateral parts of the breast. The US examinations were performed by board-certified radiographers classified by the ACR BI-RADS US standard.

The location and size of the lesions detected by MG and US were recorded. Lesion location was classified as located in the upper outer quadrant of the breast, the lower outer quadrant, the upper inner quadrant, the lower inner quadrant, the breast areola region, or the axillary tail region. Lesion size was classified as ≤2.0 cm, 2.1 to 5.0 cm, or >5.0 cm.

### Surgery

All included patients underwent surgery. The location and size of the lesions were recorded during the surgery according to the same standard as MG and US. Pathology results were collected.

### Data collection

Data were collected including BI-RADS category, microcalcifications, menstrual status, histopathology, lesion size, breast density, and breast volume. For the purpose of the present study, BI-RADS MG and US categories 1, 2, and 3 were considered as negative, and categories 4 and 5 were considered as positive.

### Statistical analysis

SPSS 16.0 (SPSS Inc., Chicago, IL, USA) was used for statistical analysis. The breast cancer sensitivity, specificity, accuracy, false-positive, false-negative, positive predictive value, and negative predictive value were calculated. Histopathological examination was considered as the gold standard. A true negative was defined as negative benign lesion by histopathology. A true positive was defined as positive evidence of malignancy on histopathology. BI-RADS categories of 0 were excluded from sensitivity, specificity, accuracy, false-positive, false-negative, positive predictive value, and negative predictive value analysis but were kept for the analysis of the location agreement. Lesion size and location were compared between imaging modalities and surgery.

## Results

### Characteristics of the patients

Of the 274 patients, 132 were with pathologically proven malignancy and 142 were benign. Among these patients, 185 (67.5%) were premenopausal and 89 (32.5%) were postmenopausal. Patients aged from 24 to 80 years, with 129 (47.1%) being ≤45 years old and 145 (52.9%) being >45 years old. The clinical data are shown in Table 1.

**Table 1:** **Patients’ characteristics**

**Characteristics**	***N*** **(%)**
Age	
≤45	129 (47.1)
>45	145 (52.9)
Menstrual status	
Premenopausal	185 (67.5)
Postmenopausal	89 (32.5)
Pathology	
IDC	120 (43.8)
DCIS	7 (2.6)
Fibroadenoma	47 (17.2)
Papilloma	9 (3.3)
Adenosis	50 (18.2)
Inflammation	14 (5)
Lipomyma	7 (2.6)
Cyst	8 (2.9)
Others (malignant)	5 (1.8)
Others (benign)	7 (2.6)
Lesion size (cm)	
≤2	122 (44.5)
2.1 to 5	135 (49.3)
>5	17 (6.2)
Breast Density	
ACR1	41 (15)
ACR2	92 (33.6)
ACR3	127 (46.3)
ACR4	14 (5.1)
Breast Volume (ml)	
≤400	120 (43.8)
400 to 800	142 (51.8)
>800	12 (4.4)

ACR. American College of Radiology; DCIS, ductal carcinoma in situ; IDC, invasive ductal carcinoma.

### Comparison between MG and US assessment

As shown in Table 1, 41 (15.0%) cases were classified as ACR level 1; 92 (33.6%) were level 2; 127 (46.3%) were level 3; and 14 (5.1%) were level 4. The average breast volume of the 274 cases was 419 ± 149 ml (range 91 to 1,130 ml), among whom 120 (43.8%) were ≤400 ml, 142 (51.8%) were 400 to 800 ml, and 12 (4.4%) were >800 ml.

Of the 274 cases, MG BI-RADS category was 0 in 38 (13.9%) cases, category 1 in 30 (10.9%), category 2 in 7 (2.6%), category 3 in 43 (15.7%), category 4 in 113 (41.2%), and category 5 in 43 (15.7%). US BI-RADS category was 1 in 4 (1.5%) cases, category 2 in 32 (11.7%), category 3 in 67 (24.5%), category 4 in 150 (54.7%), and category 5 in 21 (7.7%) (Table 2).

**Table 2:** **BI-RADS categories in mammography and ultrasonography**

**BI-RADS**	**MG**	**US**
0	38 (13.9%)	0 (0%)
1	30 (10.9%)	4 (1.5%)
2	7 (2.6%)	32 (11.7%)
3	43 (15.7%)	67 (24.5%)
4	113 (41.2%)	150 (54.7%)
5	43 (15.7%)	21 (7.7%)
**Total**	274 (100%)	274 (100%)

BI-RADS, Breast Imaging Reporting and Data System; MG, mammography; US, ultrasonography.

### Comparison of the diagnostic accuracy between MG and US

**Figure 1:**
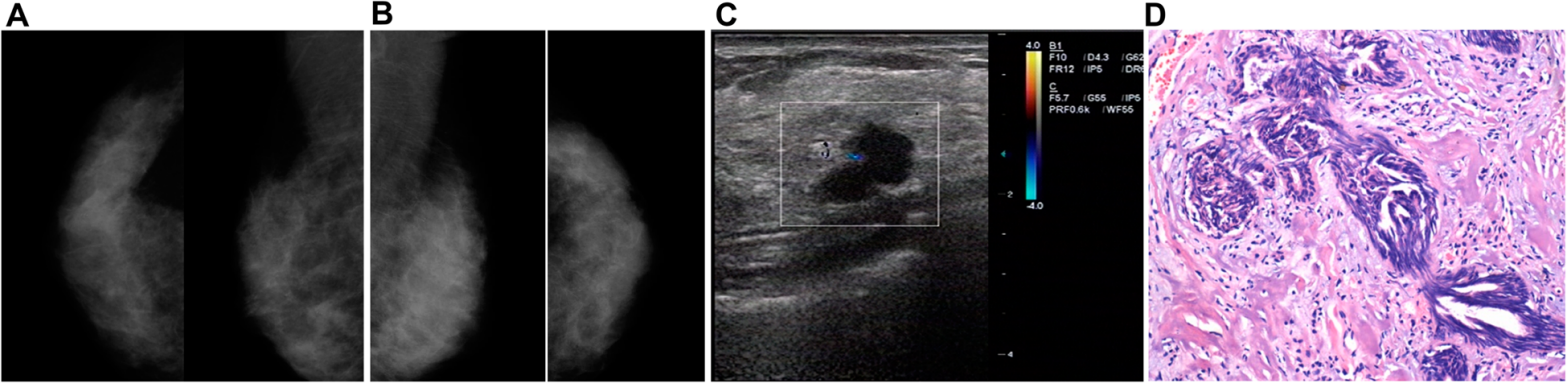
MG, US, and post-surgical pathology results from a 43-year-old patient with a lump in her right breast. **(A)** Craniocaudal and mediolateral oblique views of the molybdenum target MG of the right breast. MG were unsatisfactory (BI-RADS category 0) because of the dense gland structure. **(B)** MG of the left breast of the same patient. **(C)** US detected an irregular hypoechoic lesion with clear boundaries. **(D)** Intra-operative pathology revealed an invasive ductal carcinoma (HE staining, ×100). This is a typical example of a missed diagnosis of breast disease by MG.

The overall sensitivity, specificity, accuracy, false-positive, false-negative, positive predictive value, and negative predictive value for the detection of breast cancer were 88.5%, 57.9%, 73.7%, 42.1%, 11.5%, 69.2%, and 82.5%, respectively, for MG and 95.9%, 66.7%, 81.8%, 33.3%, 4.1%, 75.5%, and 93.8%, respectively, for US. The overall values of US were higher than that of MG. These values were then stratified according to age, menstrual status, breast density, and breast volume (Table 3). Subgroups analyses presented in Table 3 also suggest that sensitivity and accuracy were lower with MG than with US in women ≤45 years old (sensitivity: 73.7% vs. 89.5%, accuracy: 65.4% vs. 76.0%), premenopausal (sensitivity: 81.0% vs. 91.9%, accuracy: 64.9% vs. 79.7%), or with high breast density (sensitivity: 63.2% vs. 92.3%, accuracy: 71.7% vs. 79.7%). We excluded 38 (14%) patients who were classified as BI-RADS category 0 in MG because the imaging findings were unsatisfactory to detect the lesions. Among them, 7 cases were invasive ductal carcinoma (IDC). Thirty (10.9%) cases with MG BI-RADS category 1 underwent surgery because of the presence of an obvious mass by US or clinical examination. Among these 30 patients, histopathology results revealed the presence of 9 cancers and 21 benign lesions. Figure 1 shows a typical example of the missed diagnosis of breast disease by MG.

**Table 3:** **Comparison of diagnostic value between MG and US**

	**Method**	**Sensitivity**	**Specificity**	**Accuracy**	**False-positive**	**False-negative**	**Positive PV**	**Negative PV**
All	MG	88.5%	57.9%	73.7%	42.1%	11.5%	69.2%	82.5%
	US	95.9%	66.7%	81.8%	33.3%	4.1%	75.5%	93.8%
Age ≤45 years	MG	73.7%	60.6%	65.4%	39.4%	26.3%	51.9%	80.0%
	US	89.5%	68.2%	76.0%	31.8%	10.5%	61.8%	91.8%
Age >45 years	MG	95.2%	54.2%	80.3%	45.8%	4.8%	78.4%	86.7%
	US	98.8%	64.6%	86.4%	35.4%	1.2%	83.0%	96.9%
Premenopausal	MG	81.0%	53.8%	64.9%	46.2%	19.0%	54.8%	80.3%
	US	91.9%	71.4%	79.7%	28.6%	8.1%	68.7%	92.9%
Postmenopausal	MG	96.6%	73.9%	90.2%	26.1%	3.4%	90.5%	89.5%
	US	100.0%	47.8%	85.4%	52.2%	0.0%	83.1%	100.0%
Low breast density	MG	92.9%	52.1%	76.3%	47.9%	7.1%	73.9%	83.3%
	US	98.6%	62.5%	83.9%	37.5%	1.4%	79.3%	96.8%
High breast density	MG	63.2%	82.7%	71.7%	17.3%	36.8%	82.7%	63.2%
	US	92.3%	69.7%	79.7%	30.3%	7.7%	70.6%	92.0%

MG, mammography; PV, predictive value; US, ultrasonography.

### Comparison of agreement rate with surgery between MG and US

**Figure 2:**
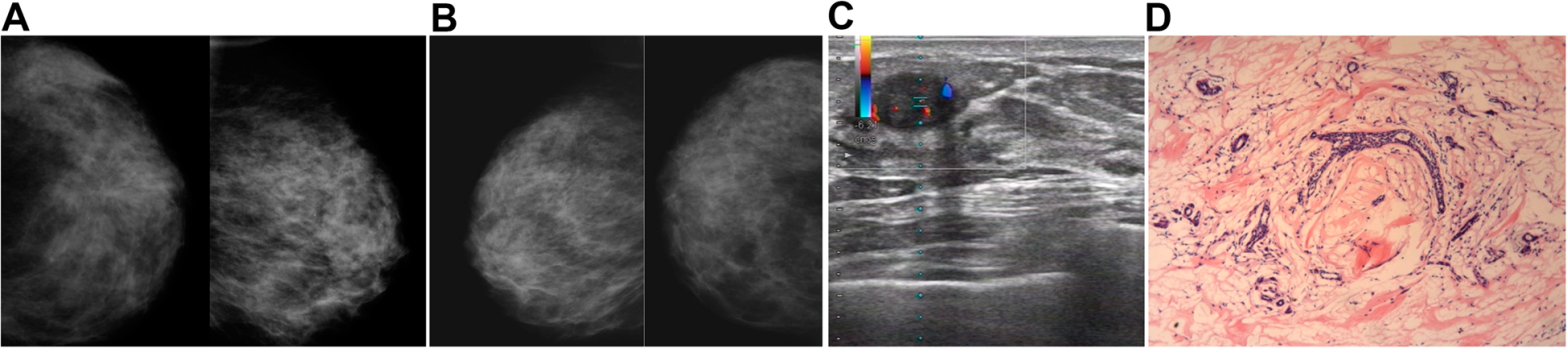
MG, US, and post-surgical pathology results from a 46-year-old patient with a lump in her left breast. **(A)** MG of the left breast could not identify the location of the lesions. **(B)** MG of the right breast of the same patient. **(C)** US showed an oval mass in her left breast. **(D)** Post-surgical pathological examination revealed adenosis of the left breast complicated by fibroadenoma (HE staining, ×100). This represents a typical example of misdiagnosed lesion sites by MG compared with pathological examination.

**Figure 3:**
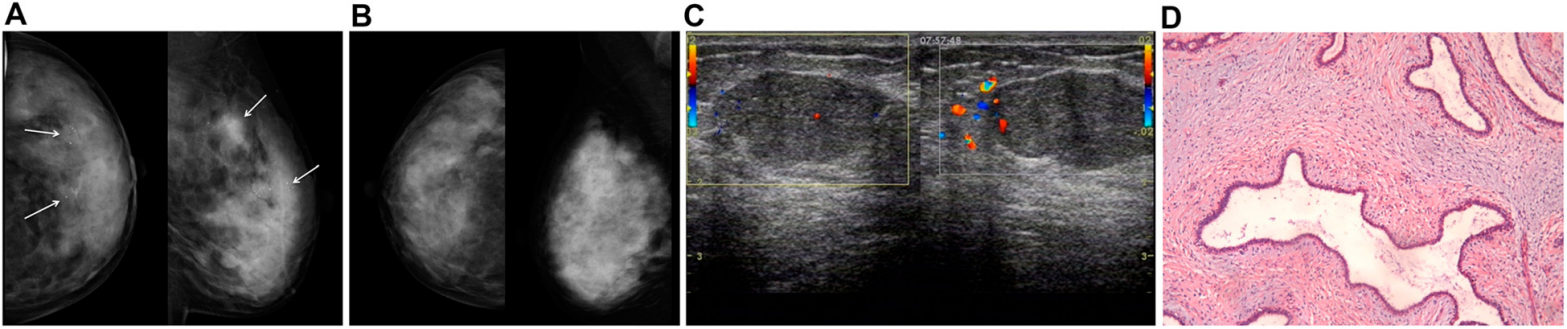
MG, US, and post-surgical pathology results from a 38-year-old patient with a lump in her left breast. **(A)** Craniocaudal and mediolateral oblique MG of the left breast displayed lesions in the left outer breast, with multiple clusters of microcalcifications, unclear lesion boundaries (white arrows), and unknown lesion sizes. **(B)** MG of the right breast of the same patient. **(C)** The size of lesion was 3 cm by US. **(D)** Post-surgical pathological examination revealed adenosis of the breast complicated by fibroadenoma, with focal calcifications (HE staining, ×100). This represents a typical example of the inability of MG to correctly determine the lesion boundaries and size compared with pathological examination.

Of the 274 cases, lesion size by MG agreed with surgery in 131 (47.8%) patients compared with 217 (79.2%) by US. Of the 274 cases, lesion location by MG agreed with surgery in 128 (46.7%) patients compared with 250 (91.2%) by US. These values were then stratified according to age, menstrual status, breast density, and breast volume (Table 4), and the agreement rates of MG with surgery were lower than that of US (all *P* < 0.01), except when the lesion size was >5 cm (*P* > 0.05) (Table 4). As shown in Figures 2 and 3, MG often failed to identify the size and location of the lesion due to dense glands and overlapping structures.

**Table 4:** **Comparison between MG and US for agreement rates for lesion size and location**

**Variable**	**Lesion size**			**Lesion location**		
	**MG**	**US**	***P*** **value**	**MG**	**US**	***P*** **value**
All	131/274 (47.8%)	217/274 (79.2%)	<0.001	128/274 (46.7%)	250/274 (91.2%)	<0.001
Age ≤45	55/129 (42.6%)	100/129 (77.5%)	<0.001	52/129 (40.3%)	116/129 (89.9%)	<0.001
Age >45	76/145 (52.4%)	117/145 (80.7%)	<0.001	76/145 (52.4%)	134/145 (92.4%)	<0.001
Premenopausal	82/185 (44.3%)	144/185 (77.8%)	<0.001	81/185 (43.8%)	167/185 (90.3%)	<0.001
Postmenopausal	49/89 (55.1%)	73/89 (82%)	<0.001	47/89 (52.8%)	83/89 (93.3%)	<0.001
Low breast density	74/133 (55.6%)	109/133 (82.0%)	<0.001	67/133 (50.4%)	123/133 (92.5%)	<0.001
High breast density	57/141 (40.4%)	108/141 (76.6%)	<0.001	61/141 (43.3%)	127/141 (90.1%)	<0.001
Lesion <2 cm	46/122 (37.7%)	102/122 (83.6%)	<0.001	43/122 (35.2%)	111/122 (91.0%)	<0.001
Lesion 2.1 to 5 cm	79/135 (58.5%)	108/135 (80.0%)	<0.001	74/135 (54.8%)	123/135 (91.1%)	<0.001
Lesion >5 cm	6/17 (35.3%)	7/17 (41.2%)	0.724	11/17 (64.7%)	16/17 (94.1%)	0.09

MG, mammography; US, ultrasonography.

The chi-square test was used for agreement rates for lesion size and location between MG and US. *P* values <0.05 were considered as significant.

## Discussion

The aim of the present study was to compare X-ray MG and US in the diagnosis of breast diseases in Chinese women. Results showed that the overall sensitivity, specificity, accuracy, false-positive, false-negative. positive predictive value, and negative predictive value were significantly higher with US than with MG. Subgroups analyses suggested that sensitivity and accuracy were lower with MG than with US in women ≤45 years old, premenopausal, or with high breast density. Compared with the surgical data, the agreement rates for lesion size and location of MG were lower than that of US (all *P* < 0.01), except when the lesion size was >5 cm (*P* > 0.05). These results suggest that US could be a better breast imaging modality for Chinese women.

Assessment of breast diseases with imaging modalities such as MG and US provides a mean for lesion detection and diagnosis. In western countries, MG is the primary breast cancer screening tool and has demonstrated evidences of reduction of breast cancer mortality [9-11]. However, compared with women from western countries, Chinese women have their unique characteristics such as high breast density and small breast volume that influence the sensitivity and accuracy of MG in detecting breast diseases [12]. Breast density is negatively associated with MG sensitivity [13], as well as with mortality from breast cancer [14]. Indeed, the intrinsic limitations of MG result in failure to detect 10% to 15% of breast cancers, and MG sensitivity is reduced particularly in women with dense breast tissue [1], as shown in the present study. These data suggest that MG might not be an optimal choice for detecting breast lesions in Chinese women [15,16], which is supported by a study performed in American women with dense breasts [17,18].

In the present study, all patients were from the Anhui Province, which is an undeveloped province in the middle of China, and most of these patients had dense breast tissue and small breast volume. Of the 274 cases, 38 (13.9%) were classified as BI-RADS category 0, meaning that an important proportion of women undergoing MG could not be satisfactorily assessed, which is supported by previous studies [19,20]. In addition, 30 (10.9%) patients assessed as being BI-RADS category 1 by MG had a palpable mass by clinical examination or had an obvious mass by US, prompting surgery. Among these 30 patients, nine were diagnosed with cancer. Therefore, these results suggest that even MG BI-RADS category 1 was not accurate enough and may miss some malignant lesions.

In the present study, MG had significantly lower sensitivity, specificity, accuracy, false-positive, false-negative, positive predictive value, and negative predictive value than US. Stratified analysis showed that young age, premenopausal, and high breast density decreased the diagnostic accuracy of MG. Indeed, dense breast tissues interfere with the interpretation of MG [5,20-23].

In addition, MG could not exactly determine the size and location of the breast lesion in many cases. This method only achieved a low agreement rate with surgery for detecting the lesion size and location. A potential reason is that the surrounding tissues and the lesions have similar X-ray attenuation, covering the shape and size of the mass. Therefore, some small cancers may be missed, and some benign lesions may be subjected to an unnecessary surgery. Nevertheless, MG showed good sensitivity for large palpable lesions, but these lesions would undergo surgery anyway.

Compared with MG, dense breast tissues are hyperechoic on US and most lesions are hypoechoic [24,25]. Therefore, because US are not affected by high density breast tissues, breast US has a higher sensitivity for detecting breast cancers in women with dense breast tissue [26-28]. Therefore, since Chinese women often have dense breasts, US should be more effective, accurate, and useful as the breast imaging tools. In addition, women are not exposed to radiations.

In the present study, the results strongly suggest that US was significantly better than MG for detecting breast diseases. There was no BI-RADS category 0 case reported by US. In young women and women with dense breasts, US appears superior to MG as an effective diagnostic tool in the evaluation of breast diseases. US had a significantly greater diagnostic accuracy than MG. Finally, US had a high agreement rate with surgery and it could be used to determine the exact size and location of the breast lesions. Therefore, US could be a better screening modality than MG in Chinese women. In addition, it is much cheaper than other modalities such as MRI, making it the modality of choice for areas with a poor economic status. MRI’s sensitivity to invasive cancers is nearly 100% [29-31], and that it is not influenced by age or gland density degree [31,32]. However, MRI is not the best imaging modality to assess microcalcifications detected on MG since MRI is based on changes in the spin of hydrogen protons and that microcalcifications contain few of these [33]. In addition, MRI machines are expensive, as well as the examinations *per se*. Nevertheless, US should be compared with new modalities such as breast tomosynthesis [34,35]. In some centers, MG and US could be used together to maximize the detection of breast cancer [36]. In young asymptomatic high-risk women (<50 years old), digital MG could be used as the primary screening modality, and US could be performed if necessary [37]. These results could be generalized to all women with dense breasts, not only Chinese ones.

The present study is not without limitations. In addition to its retrospective nature, the sample size was small and was from a single center. Multicenter studies should be performed to confirm these results.

## Conclusions

In conclusion, US was better than MG in the preoperative evaluation of breast diseases of Chinese women. These results suggest that US could be more useful for detecting breast lesions in China.

## Abbreviations

MG: mammography
MRI: magnetic resonance imaging
